# DeepMalaria: Artificial Intelligence Driven Discovery of Potent Antiplasmodials

**DOI:** 10.3389/fphar.2019.01526

**Published:** 2020-01-15

**Authors:** Arash Keshavarzi Arshadi, Milad Salem, Jennifer Collins, Jiann Shiun Yuan, Debopam Chakrabarti

**Affiliations:** ^1^Burnett School of Biomedical Sciences, University of Central Florida, Orlando, FL, United States; ^2^Department of Electrical and Computer Engineering, University of Central Florida, Orlando, FL, United States

**Keywords:** artificial intelligence, malaria, drug discovery, virtual screening, deep learning, inhibition, toxicity

## Abstract

Antimalarial drugs are becoming less effective due to the emergence of drug resistance. Resistance has been reported for all available malaria drugs, including artemisinin, thus creating a perpetual need for alternative drug candidates. The traditional drug discovery approach of high throughput screening (HTS) of large compound libraries for identification of new drug leads is time-consuming and resource intensive. While virtual *in silico* screening is a solution to this problem, however, the generalization of the models is not ideal. Artificial intelligence (AI), utilizing either structure-based or ligand-based approaches, has demonstrated highly accurate performances in the field of chemical property prediction. Leveraging the existing data, AI would be a suitable alternative to blind-search HTS or fingerprint-based virtual screening. The AI model would learn patterns within the data and help to search for hit compounds efficiently. In this work, we introduce DeepMalaria, a deep-learning based process capable of predicting the anti-*Plasmodium falciparum* inhibitory properties of compounds using their SMILES. A graph-based model is trained on 13,446 publicly available antiplasmodial hit compounds from GlaxoSmithKline (GSK) dataset that are currently being used to find novel drug candidates for malaria. We validated this model by predicting hit compounds from a macrocyclic compound library and already approved drugs that are used for repurposing. We have chosen macrocyclic compounds as these ligand-binding structures are underexplored in malaria drug discovery. The *in silico* pipeline for this process also consists of additional validation of an in-house independent dataset consisting mostly of natural product compounds. Transfer learning from a large dataset was leveraged to improve the performance of the deep learning model. To validate the DeepMalaria generated hits, we used a commonly used SYBR Green I fluorescence assay based phenotypic screening. DeepMalaria was able to detect all the compounds with nanomolar activity and 87.5% of the compounds with greater than 50% inhibition. Further experiments to reveal the compounds’ mechanism of action have shown that not only does one of the hit compounds, DC-9237, inhibits all asexual stages of *Plasmodium falciparum*, but is a fast-acting compound which makes it a strong candidate for further optimization.

## Introduction

Malaria is one the deadliest disease afflicting the mankind, with more than 200 million new cases every year, and over 400,000 reported deaths (WHO, 2018). The causative agent of infection, *Plasmodium spp*. parasites have developed resistance to almost all currently marketed drugs including the current treatment choice artemisinin-based combination therapy (ACT) (Fairhurst and Dondorp, 2016). This underscores an urgent need to discover next generation antimalarials (Cowell and Winzeler, 2019). Traditionally, the discovery of new bioactive chemotypes relies on cell or target-based screening (Baniecki et al., 2007) (Swinney, 2013) of natural or synthetic compound libraries. High Throughput Screening (HTS) using either approach entails screening of large library of compounds. This process is often inefficient and not cost effective because of high failure rate at subsequent stages of drug discovery. The real question is, with all the modern technological advancements in drug discovery how can we utilize innovative technologies to find new active compounds more efficiently, thus reducing the cost?

Screening of large diverse compound libraries is likely to yield a higher hit rate. The bioactivity of a compound can also be predicted *in silico* through virtual screening (Shoichet, 2004). In this approach, models are created to predict the activity of a compound based on chemical properties of the compounds. One of the most common descriptors currently used for virtual screening is Extended Connectivity Fingerprint (ECFP) (Rogers and Hahn, 2010). The ECFP uses topological characteristics of a molecule to describe it. The most prevalent use of ECFP in Quantitative Structure-Activity Relationship (QSAR) models involves creating a fingerprint and using a neural network to perform prediction (Ramsundar et al., 2015; Gupta et al., 2016). This approach isolates feature extraction and decision making, thus not allowing the decision-making process to have an effect on the creation of fingerprints.

With the availability of large datasets, such as whole genome sequencing, transcript profiling or HTS, artificial intelligence is expected to have major impacts on various aspects of biomedical research (Jiang et al., 2017; Wainberg et al., 2018; Reddy et al., 2019; Zhavoronkov et al., 2019). Application of AI to various areas of drug discovery would include ligand-based virtual screening (VS) (Mayr et al., 2016; Chen et al., 2018), target prediction (Mayr et al., 2018), structure-based virtual screening (Wallach et al., 2015), de novo molecular design (Kadurin, 2016; Aspuru-Guzik, 2018), or metabolomics approaches (Pirhaji et al., 2016). Deep learning approaches enable end-to-end classification of data via learning feature representation and decision making simultaneously. Deep learning’s automatic feature extraction has demonstrated superiority to traditional isolated feature extraction and has resulted in the popularity of these models in many fields such as image recognition, signal classification (Rajpurkar, 2017), and deep processing of natural language (Devlin, 2019).

Recently, Graph Convolutional Neural Networks (GCNN) have shown high accuracy in predicting chemical properties of compounds (Aspuru-Guzik et al., 2015). These models transform the compounds into graphs and learn higher-level abstract representations of the input solely based on the data. Graph convolutional neural networks combine ECFP’s concept of creating fingerprints from substructures with deep learning’s automatic feature extraction. Compared to ECFP, the GCNN’s features are shorter (encoding only the relevant features), contain similarity information for different substructures, and facilitate more accurate predictions (Aspuru-Guzik et al., 2015; Kearnes et al., 2016; Liu et al., 2018).

In this work, we leverage GCNNs to accelerate the process of antimalarial drug discovery. The representative abilities of GCNNs are used to implement a virtual screening pipeline. These models take compounds as input and predict the *P. falciparum* growth inhibition and mammalian HepG2 cell cytotoxicity of the given compounds, aiding in the intelligent selection of scaffolds as input for further analysis. The hyper-parameters of the model are optimized using an external validation on an independent and imbalanced dataset. To overcome the difficulty of low training data, transfer learning is used. The model is initialized with the weights transferred from a model trained on a large unrelated dataset. The compounds are further tested using *in vitro* bioassay for validation of the model.

Another area of drug discovery which increases the probability of detecting high value scaffolds would be the selection of compound libraries. Principal component analysis of about 5 million compounds screened against the malaria parasite *Plasmodium falciparum* in last ten years suggests that not only the libraries used have low diversity but also, they mostly consist of compounds with low molecular weight (Spangenberg et al., 2013). Drug discovery efforts in last few decades using Lipinski “rule of five” compliant synthetic compound libraries are exhibiting diminishing return. Furthermore, biological targets of approved drugs are quite limited. Therefore, for our analysis, we decided to use an unexplored natural product (NP)-inspired class of molecules. NP or NP-inspired compounds have made tremendous impacts in discovery of novel drugs (Butler et al., 2014; Newman and Cragg, 2016). Among the NPs, macrocycles have successful record as efficacious compounds with more than 100 approved drugs in the market (Blanco, 2019). At least 3% of 100,000 NP secondary metabolites are macrocycles (Driggers et al., 2008). Macrocycles are scaffolds with a ring containing at least 12 atoms (Mallinson and Collins, 2012). Macrocycles also contain many desirable properties such as, less rigidity and flexibility, high binding capabilities, having affinity to anions and cations, high bio-availability, and the ability to target protein-protein interactions (PPI) (Choi and Hamilton, 2003; Dougherty et al., 2017; Ermert, 2017; Selwood, 2017).

We present here the application of GCNNs for non-targeted ligand-based virtual screening for antimalarial drug discovery. Our research described in this article creates a practical pipeline for training generalizable virtual screening models, and the use of deep learning techniques such as transfer learning and external validation to improve the model. Results of the model to discover antiplasmodial scaffolds were validated in a prospective manner via comparison to whole cell screening.

## MaterialS and Methods

### Data

#### Training Data

GlaxoSmithKline group tested around two million compounds for inhibition of *Plasmodium falciparum* (Pf) Dd2, a chloroquine resistant line, intraerythrocytic life cycle and identified 13,533 bioactive compounds that exhibited greater than 80% inhibition of the *in vitro* growth of the parasite at 2 µM concentrations. This published data are publicly available in the supplementary material 1 of the article (Gamo et al., 2010). DeepMalaria uses this Pf Dd2 inhibition and selectivity data for training. The molecules are classified as one if they possess Dd2 growth inhibitions of 50% and higher and zero if otherwise. The efficacy of these compounds differs in *P. falciparum* strains Dd2 and 3D7 (a *Pf* line sensitive to chloroquine), with most of the molecules in the GSK dataset possessing higher 3D7 inhibition and varying Dd2 inhibition. Therefore, the training data implicitly holds information about the developed resistance, and if the model is trained on Dd2 inhibition data, it would be able to predict compounds that are efficacious in drug resistant strains, a desirable property.

#### Validation Data

The validation dataset consists of the results from previously performed HTS in University of Central Florida at the Chakrabarti Laboratory, consisting of natural-products, kinase inhibitors from commercial vendors Asinex (Winston-Salem, NC)and ChemDiv (San Diego, CA) libraries. This dataset contains 4,497 molecules and their inhibition property. Overall, the dataset possesses 112 molecules that have a Pf inhibition greater than 50%. Using this external validation dataset, the realistic capabilities of the model are evaluated in the validation process. The raw data supporting the conclusions of this manuscript will be made available by the authors, without any restriction, to any qualified researcher (**Supplementary Material 4**).

#### Compound Library for Test Data

A library of 2,400 macrocyclic compounds was purchased from the commercial vendor Asinex (Winston-Salem, NC) for validation. The compounds selected for purchase were not given any consideration about DeepMalaria prediction to avoid any bias in results (**Supplementary Material 3**).

#### Source Data for Transfer Learning

In order to perform transfer learning, a large dataset is chosen as the source to transfer from. One of the largest labeled molecule datasets is publicly available in the PubChem Bioassay (PCBA) repository. Within this dataset, the “PCBA-686979” assay (Wu et al., 2018; Pubchem Database, 2019) contains 303,167 molecules with 20.82% of them being active. The molecules in the mentioned library are not related to *Plasmodium*, and they were screened to find inhibitors of human tyrosyl-DNA phosphodiesterase 1 (TDP1). This enzyme is a target for cancer therapy in spite of not being necessary protein for human cells. This unrelated large and high variance collection is chosen as the source for transfer learning solely based on its size.

### In Silico

#### Graph Convolutional Neural Network Model

In the research described here, DeepChem’s implementation of GCNN was used (Ramsundar et al., 2019). This implementation offers the creation of architectures with graph convolutional layers, graph pooling layers, dropout layers, graph gather layers, and fully connected layers. The molecular graph was sorted via atom index in order to attain the same graph for canonical SMILES. The training data was first cleaned by removing the molecules with missing inhibition data. Two details needed to be considered in the conversion of molecules to graphs; firstly, the nodes represent different atoms and need to contain information of this difference. In order to differentiate between the atom nodes, DeepChem offers 75 different features for describing each atom. In this work, 29 of those features were used containing the type of atom, atom’s degree, atom’s implicit valence, atom’s hybridization, atom’s aromatic properties, and total number of Hydrogen connected to the atom. Secondly, in order to convert molecules to graph and not lose special information, chirality was added to the features.

#### Data Augmentation and Hyper-Parameter Optimization

The validation dataset for this work, i.e., the “lab dataset” is highly imbalanced. Only 2% of the molecules within the dataset show inhibitory activity. These molecules are also the most important part of the dataset, since the goal of the model is to find active molecules. In order to have a fair validation on this dataset, the data needs to be balanced first. The data augmentation process created more copies of the active molecules after shuffling the atom orders. This balancing process is done via SMILES Enumeration (Bjerrum, 2017), creating on average 38 copies of each active molecule.

The augmented validation dataset can be used for finding the optimum topology, hyper-parameters, and epochs for training. Starting with the topology, the hyper-parameters that can be defined are the number of convolution layers, the size of each convolution layer, number of neurons in the dense layer, and the dropout of each layer. The remaining hyper-parameters that can be defined are the learning rate and batch size. To perform hyper-parameter optimization and find a fitting architecture, grid search was performed. Different values were chosen for each hyper-parameter, the model was trained on the training dataset and tested on the validation dataset. The set of hyper-parameters that has the best performance was chosen, and the architecture and variables of the model were finalized.

#### Transfer Learning

Training a deep learning model often requires a large amount of data as the algorithms contain numerous variables that are optimized during training. DeepMalaria’s training set is in the order of a few thousands, when compared to the image domain datasets it is considered to be rather small amount of data. This amount of data makes the training of the model to be sensitive to its initial weights. In order to overcome this challenge, transfer learning was used from a larger source dataset. It has been shown that the source dataset does not necessarily need to have correlation with the target dataset. The patterns within the molecules of the transfer dataset (PCBA) can help initialize the GCNN and make the training on the target dataset (GSK) to be more efficient. After the optimized architecture for model is found, the model is trained on the source dataset for 50 epochs, then the weights are saved and restored in the beginning of training on the training dataset.

#### Evaluation of the Model

In order to assess the performance of the model, evaluation metrics are needed. One evaluation metric that is commonly used for classification task is accuracy. If the model can correctly classify active compounds as active (true positive or “TP”) and inactive compounds as inactive (true negative or “TN”), it would have a high accuracy. If the model is missing the active molecules and is incorrectly classifying them as inactive (false negative or “FN”), or if the model is predicting inactive molecules to be active (false positive or “FP”), the accuracy would be decreased. **Table 1** shows these categories for the results of classification.

**Table 1:** Classification categories.

	Truly active	Truly inactive
Predicted as Active	TP	FP
Predicted as Inactive	FN	TN

With these definitions in mind, accuracy is defined as:

$$\text{Accuracy}=\frac{\text{TP}+\text{TN}}{\text{TP}+\text{TN}+\text{FP}+\text{FN}}$$

In the field of drug discovery, having a high TP and a low FN is highly important, since the purpose of the model is to predict the active molecules that are few in number. One metric that can represent the ability of the model to capture active molecules is recall, as defined below:

$$\text{Recall}=\frac{\text{TP}}{\text{TP}+\text{FN}}$$

Since the test dataset is imbalanced, accuracy would be a misleading metric. An untrained model can classify every input as inactive and still have an accuracy of nearly 97%. Furthermore, recall alone would not be enough to evaluate models in imbalanced setting because it does not contain any information of the performance of the model on the inactive molecules. To fully display model’s behavior, normalized confusion matrix is used to show the percentage of data classified as each classification category. Additionally, the Area Under the Receiver operation Characteristic Curve (ROC-AUC or AUC) is used as a fair score metric.

#### Cytotoxicity Prediction

In order to predict the cytotoxicity of the compounds, the model was trained with the same parameters used for inhibition. However, the dependent variable in the GSK dataset was changed to contain cytotoxicity information. Inhibition percentages over 50 are considered active against the human cell line (given the label 1) and otherwise considered non-active (given the label 0). Since only the active compounds with nanomolar potency against *Plasmodium falciparum* would go to next step of evaluation, the model was tested on the nanomolar active hits to predict their cytotoxicity and is prospectively evaluated.

### In Vitro

#### *Plasmodium* Growth Inhibition Assays

*P. falciparum* cultures were maintained under standard culture conditions in RPMI 1640 medium supplemented with 25 mM HEPES, pH 7.4, 26 mM NaHCO_3_, 2% dextrose, 15 mg/L hypoxanthine, 25 mg/L gentamycin, and 0.5% Albumax II maintained at 37°C in 5% CO_2_ and 95% air. Initially, a fixed concentration phenotypic screening was performed against multidrug resistant *P. falciparum* strain Dd2 (resistant to chloroquine, pyrimethamine, and mefloquine) using a SYBR Green I assay (Johnson et al., 2007; Vossen et al., 2010). An EVO-150 robotic liquid handler (Tecan, Morrisville, NC) was used to aliquot compounds at a final concentration of 1 µM followed by addition of culture to 96-well plates (Greiner, Monroe, NC) at 1% parasitemia, 2% hematocrit. Plates were incubated for 72 h at 37°C in a humidified atmosphere 5% CO_2_/95% air prior to freezing. Plates were subsequently thawed and 1X SYBR Green I was added with lysis buffer (20 mM Tris-HCl, 0.08% saponin, 5 mM EDTA, 0.8% Triton X-100). After incubation at room temperature in dark for 1 h, the fluorescent emission was measured at excition and emission wavelengths of 485 nm and 530 nm, respectively, using a BioTek Synergy neo2 (Winooski, VT) plate reader. Preliminary hits exhibiting greater than 50% inhibition were then screened to determine EC_50_ values. For EC_50_, determination compounds were serially diluted in growth medium starting at 5 µM Untreated cultures and the ones treated with chloroquine at 1 µM rved as controls. Curve fitting was performed using GraphPad Prism and EC_50_ value was determined for each compound.

#### Cytotoxicity Determination

Selectivity was determined by counter-screening against human hepatoma cell line HepG2 in a MTS (3-(4,5-Dimethylthiazol-2-yl)-5-(3-carboxymethoxyphenyl)-2-(4-sulfophenyl)-2H-tetrazolium) based cytotoxicity assay (CellTiter 96® Aqueous One, Promega, Madison, WI) (Riss et al., 2004). Briefly, microtiter plates were seeded with 1,500 cells per well in a 384 well plate and incubated for 24 h at 37°C in 5% CO_2_/95% air atmosphere. The next day, compounds were added at seven different serially diluted concentrations starting at 25 µM d incubated for additional 48 h at stated conditions. MTS solution was next added to each well, incubated for additional 3 h at 37°C, and absorbance was read at 490 nm using BioTek Synergy neo 2 plate reader. Untreated cells served as control. Curve fitting was performed using GraphPad Prism and EC_50_ value was determined.

#### Stage-Specific Activity Assay

*P. falciparum* Dd2 cell line was synchronized using a combination of 5% sorbitol and magnetic column separation as described (Roberts et al., 2016). Cultures at 2% parasitemia and 2% hematocrit were added to microtiter plate wells and measurements began 6 h post-invasion (6 hpi). Hit compounds were added at 3X EC_50_ concentration at specified time points. Controls are DHA (3X EC_50_) and untreated cultures. Giemsa-stained thin smears were made for each time point and an aliquot of the culture was fixed for flow cytometric analysis using 0.04% glutaraldehyde in PBS. After fixing and aspirating, cells were permeabilized with 0.25% Triton-100 followed by treatment with RNase (0.05 mg/ml) for 3 h. Next, YOYO 1 (10.24 µM) fluorescent dye was added and samples were analyzed (Bouillon et al., 2013) using Beckman Coulter (Indianapolis, IN) CytoFlex S flowcytometer.

#### Rate of Killing Determination

*Pf* Dd2 culture was synchronized as described (Roberts et al., 2016), plated into 24 well plates at 2% parasitemia and 2% hematocrit at 6 hpi. Compounds were added at 6, 18, or 30 hpi with a final concentration of 5X EC_50_. Each well was exposed to the inhibitor for either 6 or 12 h using dihydroartemisinin (DHA) (25 µM) and untreated culture as controls. After washing the compounds off, the media was changed twice a day. The parasitemia was tracked for 6 days after addition of compounds. Thin smears were stained with Giemsa, and parasitemia was counted using microscope.

## Results

### Overview

**Figure 1:**
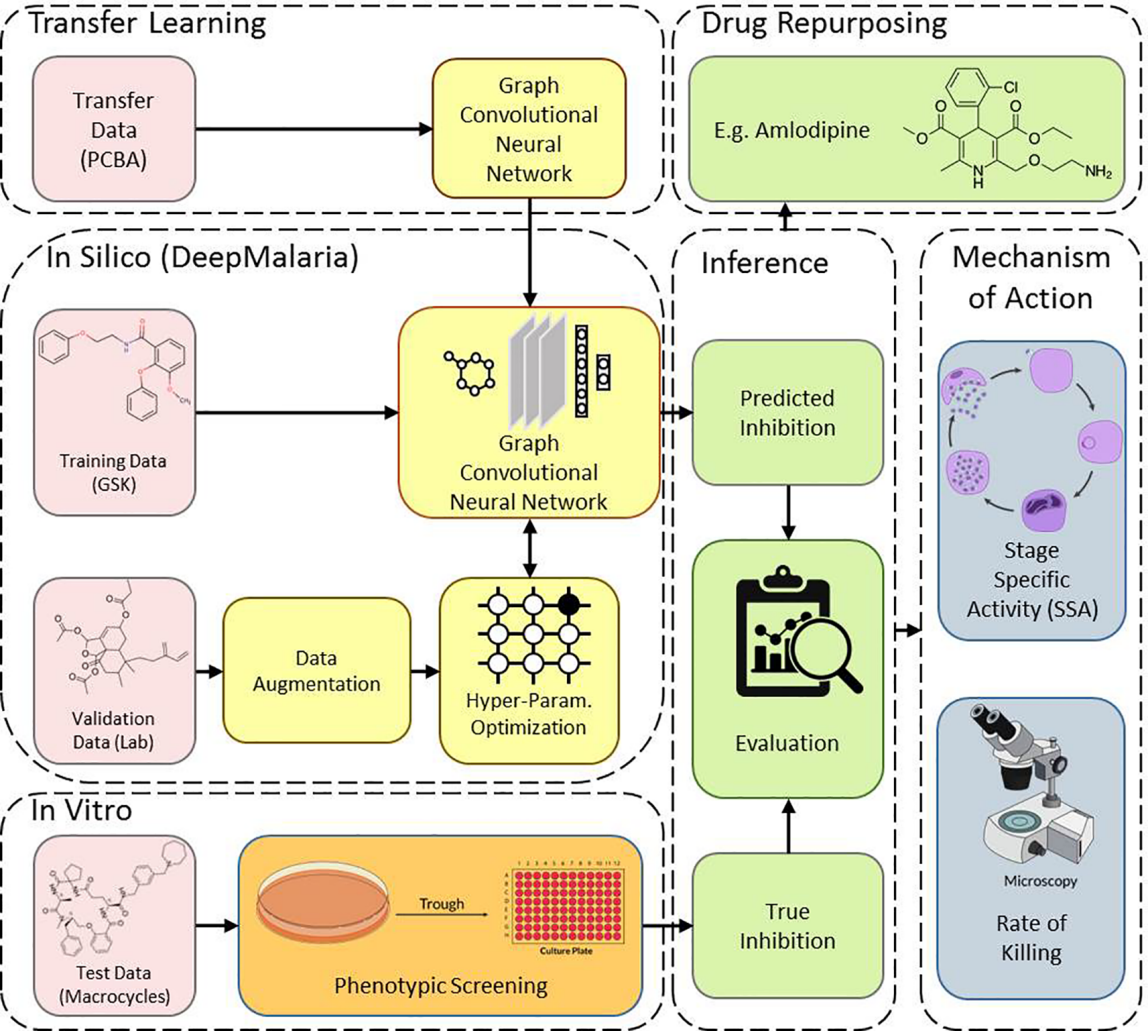
Overview of Transilico architecture used to train DeepMalaria*. In silico*, the validation dataset is augmented and is used to determine the hyper-parameters of the model. The model is pre-trained on the transfer dataset and fine-tuned on the training dataset. *In vitro*, the test compounds are tested on *Pf*. The results are compared to predictions. The trained model can be applied for drug repurposing. The mechanism of action for the hits are determined.

This work consists of two main sections: *in silico* and *in vitro*. In the *in silico* approach, DeepMalaria enables virtual screening of molecules on *Plasmodium falciparum* using a deep learning model. At the core, the GCNN model acts as a classifier, predicting the inhibition of input molecules and classifying them as “active” or “inactive.” In order to optimize the hyper-parameters of the deep learning model, the model is validated externally on an independent and augmented validation dataset. The optimized model is trained on the large transfer dataset to extract useful initialization weights from it. Then, the pre-trained GCNN model is trained on the training dataset. The overview of our method is shown in **Figure 1**. This architecture enables the use of transfer learning for in silico screening, thus we coin the term “Transilico” for it. The code for this work can be accessed through www.transilico.com.

### *In Silico* Training

**Figure 2:**
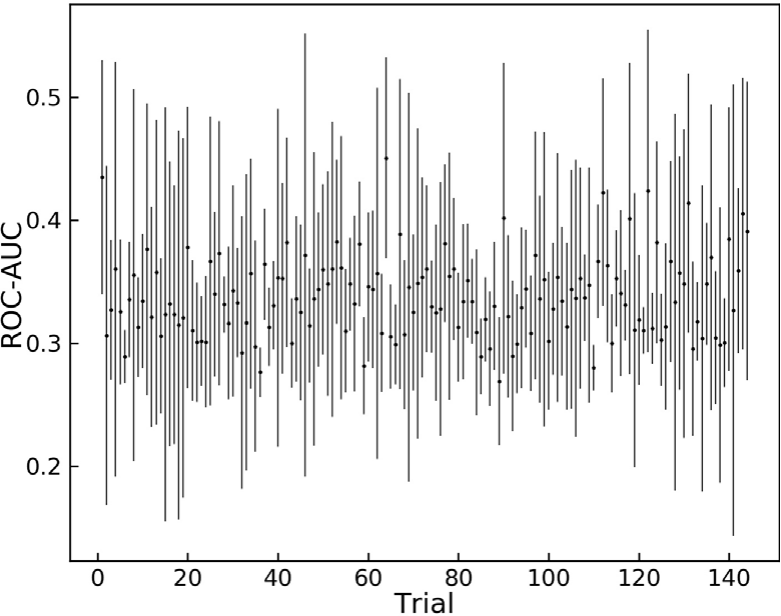
Grid search results for different sets of hyper-parameters. 144 different sets of hyper parameters for the model are tested on the augmented validation dataset.

The results of the grid search for hyper-parameter optimization are shown in **Figure 2**. Overall, 144 different combinations of hyper-parameters were chosen for training and the trained model was tested on the validation dataset.

Trial 121 is among the hyper-parameters that yielded high average ROC-AUC scores, and it achieves the highest score between all trials. These hyper-parameters were chosen as the optimum variables and are shown in **Table 2**.

**Table 2:** Finalized hyper-parameters from grid search.

Hyper-Parameter	Optimum Value	Hyper-Parameter	Optimum Value
# of Conv. Layers	3	Dropout	0
Conv. Layer Sizes	64, 64, 64	Learning Rate	0.0001
# of Neurons	256	Batch Size	128

**Figure 3:**
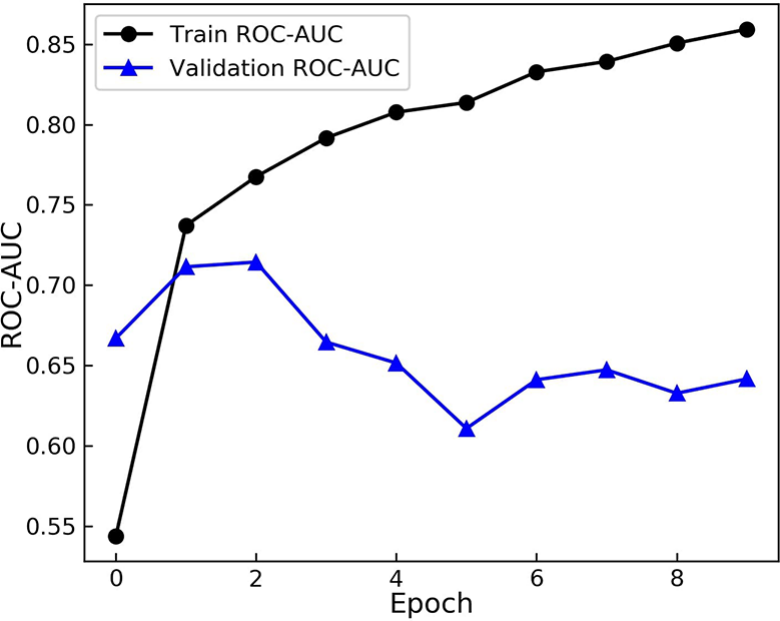
Area Under the Receiver operation Characteristic Curve (AUC) scores of the model during training. The model is evaluated on the training and augmented validation dataset at the end of each epoch. The model starts to over-fit after 2 epochs.

Having defined the architecture of the GCNN model, the model was trained on the transfer dataset. The weights were then saved and loaded for the main training process to start. The pre-trained model was fine-tuned on the training dataset with the batch size of 32. At each epoch the AUC score on the training set and the validation set were calculated and recorded. The results are shown in **Figure 3**.

As evident from **Figure 3**, the model starts to perform differently on the validation set from the training set after the 2^nd^ epoch. While the score on the training set rises and model learns the training set more, the performance on the validation dataset drops. These results demonstrate over fitting happening after the 2^nd^ epoch. Therefore, the model from this epoch is loaded as the trained model and the optimum duration of training is found.

### Phenotypic Screening Identifies Selective Compounds

Evaluation of the model was performed by phenotypic testing of compounds from a commercial macrocyclic compound library. This NP-inspired library is considered a bridge between small compounds and biomolecules (Driggers et al., 2008), thus increasing the possibility of targeting unknown biomolecules in *Plasmodium*. To rule out any validation bias in this experiment, we did not consider the *in-silico* results when buying the library, and all compounds were purchased based on their druggability as identified using traditional cluster analysis (data not shown). To compare the predictions from the DeepMalaria with the outcome of a traditional *Plasmodium* phenotypic cell-based screening, all 2,400 compounds were tested for antiplasmodial activities using SYBR green I fluorescence assay. Multi-drug resistant *Pf* Dd2 strain was used to provide clinically relevant results. Of the 2,400 compounds, 49 compounds exhibited growth inhibition of greater than 50% at 1 µM. This is a comparatively high hit rate (~2%) and provides evidence for the potential of macrocycles as a new class of antimalarial compounds. The 49 hits underwent EC_50_ determination and five compounds showed activity under 1 µM (**Table 4**). DC-9235, DC-9239, and DC-9236 are analogs and considered amino acid macrocyclic scaffolds which are novel antimalarial candidates, and further hit to lead development would increase the potency of core structure. Other compounds are not analogs of each other, suggesting discovery of four unique antimalarial macrocyclic scaffolds. The selectivity of the compounds for malaria parasite was determined by counterscreening against human hepatoma HepG2 cell line using MTS proliferation assay. All 6 hits exhibited greater than 15-fold selectivity index suggesting none is consider to be cytotoxic for HepG2 cell line (**Table 4**).

### *In Silico* and *In Vitro* Results Are Comparable

After *in vitro* phenotypic screening, the ground truth labels for the test dataset are found. The model can now be evaluated both retrospectively and prospectively, via its prediction on the validation set and the test set. The results of this evaluation are shown in **Table 3**.

**Table 3:** Results of the trained model.

	# of Active	TP	TN	FP	FN	Accuracy	Recall
Validation Dataset	112	81	2620	1756	31	60.06	72.32
Test Dataset	49	43	1016	1335	6	44.13	87.75

**Figure 4:**
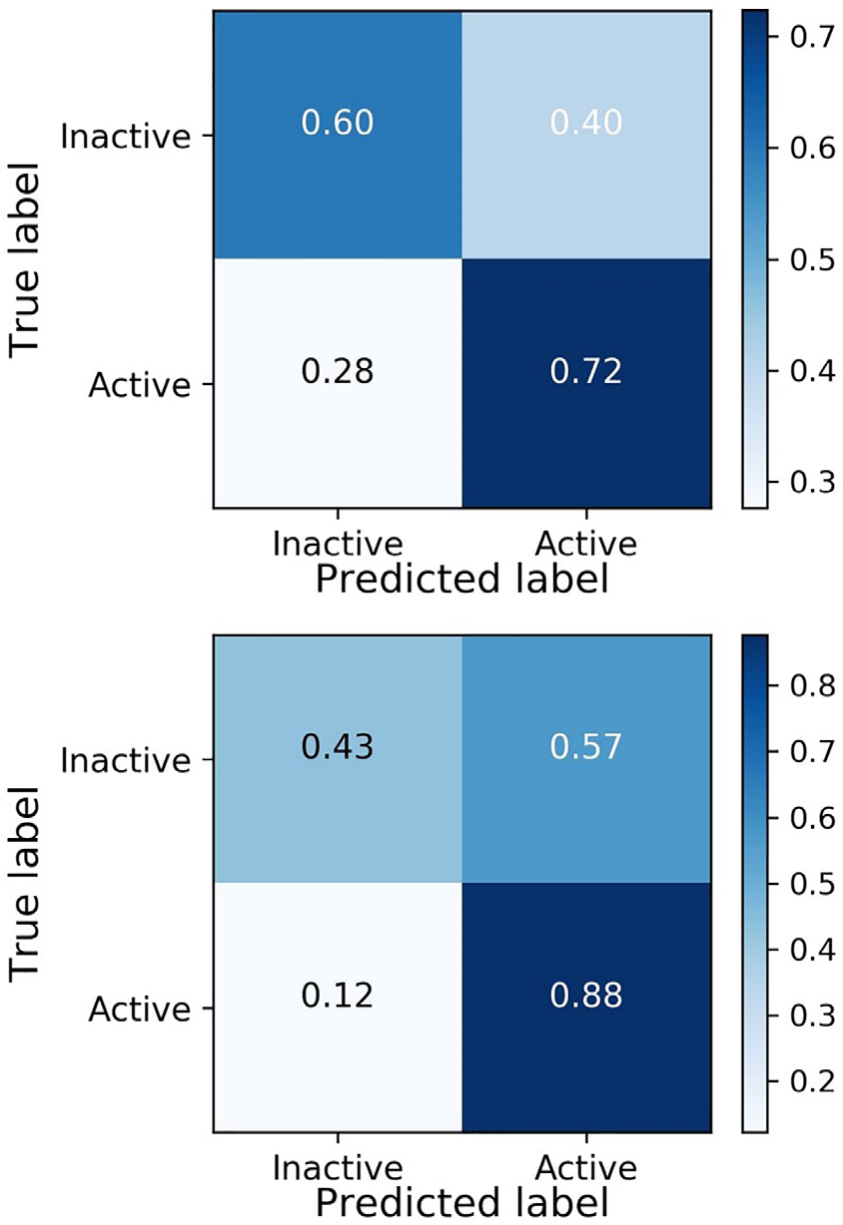
Confusion matrices of validation dataset **(A)** and test dataset **(B)**.

The model yields a high recall in both the validation and the test set, showing the ability of the model in finding active compounds. To fully display the performance of the model, the confusion matrices of the validation and test set are shown in **Figure 4**.

**Figure 4** shows similar behavior of the model on active molecules in the validation dataset and the test set, achieving the goal of the external validation process in DeepMalaria. Moreover, the model is inclined to predict the input as active, yielding a higher false positive rate than false negative rate. This behavior is essential in a drug discovery model, since finding the active molecules are of priority, and falsely predicting them as inactive will likely be counterproductive.

**Figure 5:**
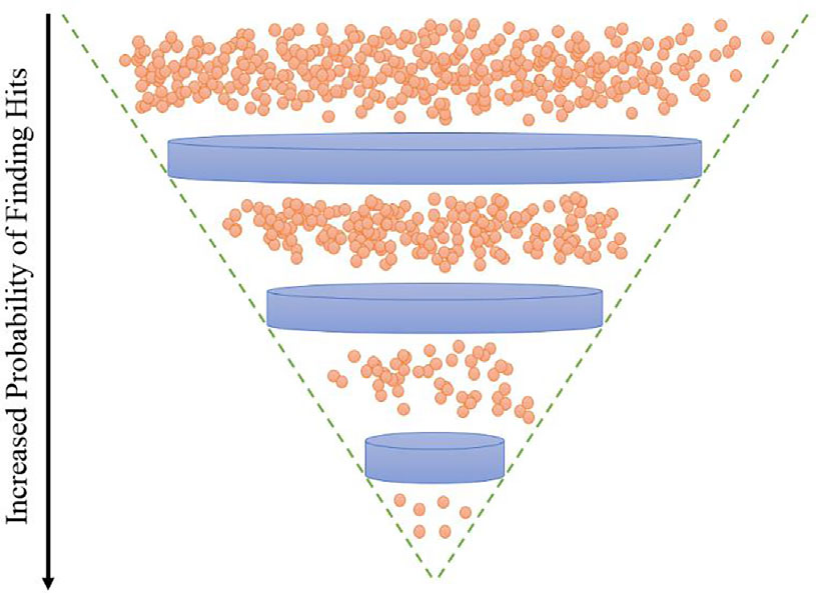
DeepMalaria finds potent hits with higher recall. 87.85% for hits with inhibition of 50% or more, 100% for nanomolar active hits.

As shown in **Table 4**, the model was able to correctly predict all of the 6 compounds with nanomolar activity. Based on the results in **Tables 3** and **4**, it is evident that DeepMalaria is capable of virtually identifying potent compounds with high accuracy. From 44.13% accuracy in the whole library, to 87.75% accuracy for hits with at least 50% growth inhibition, and finally 100% accuracy for all nanomolar active compounds, DeepMalaria is prone to have less false negative when screening more potent set of molecules. Thus, it is unlikely that the model might miss highly active compounds. (**Figure 5**). Since the identification of highly potent hit compounds is a goal of all drug discovery programs, predicting 100% of the nanomolar active hits proves the utility of AI as a rapid and low-cost alternative to traditional methods of bioactive hits discovery.

**Table 4:** *In vitro* and in silico results of the compound with Nano-molar activity.

ID	Inhibition One-point (%)	Toxicity SI	EC_50_ *µM* HepG2	EC_50_ *µM* Pf	DeepMalaria antimalarial Prediction	DeepMalaria Softmax Output	DeepMalaria Toxicity Prediction
*DC-9239*	79	25	24.2	1.09± 60nM	Active	0.70	Negative
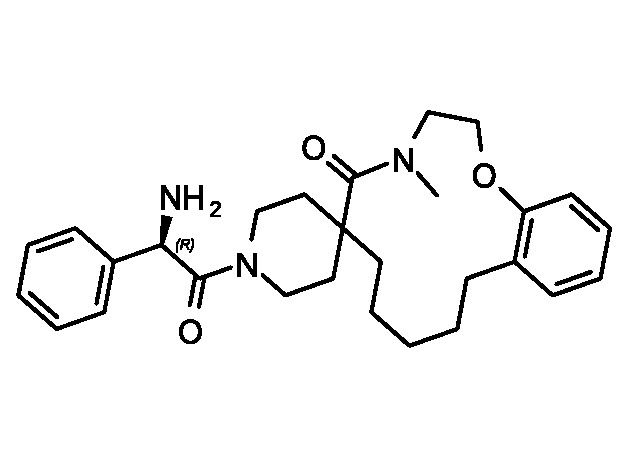
*DC-9235*	92	>40	>25	0.79± 61nM	Active	0.71	Negative
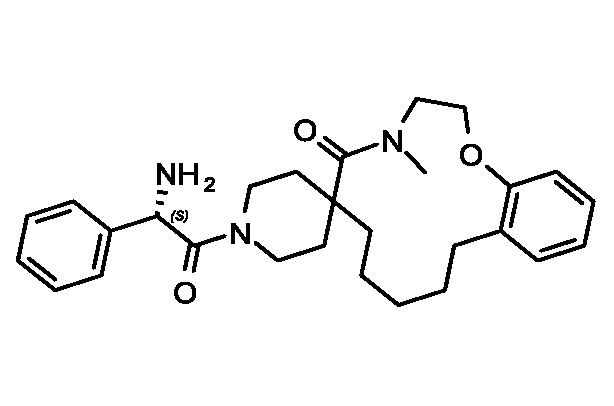
*DC-9236*	98	15.9	6.5	0.41± 30nM	Active	0.85	Negative
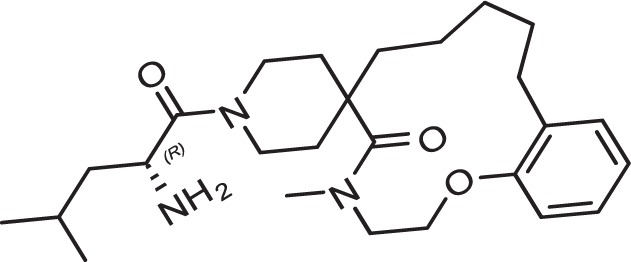
*DC-9237*	95	35.3	17	0.49± 44nM	Active	0.68	Negative
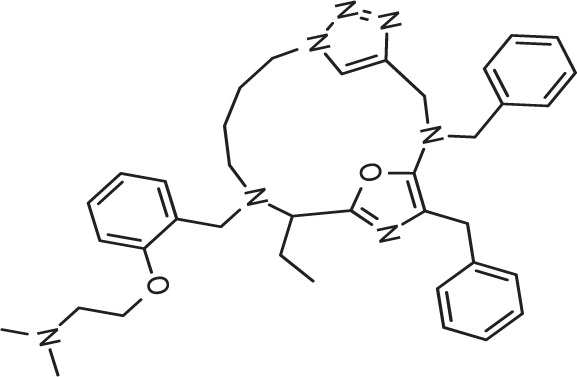
*DC-5931*	80	>40	>25	0.52± 25nM	Active	0.96	Negative
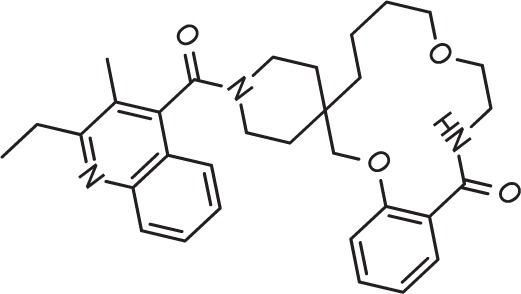
*DC-5921*	90	>40	>25	0.9± 10nM	Active	0.91	Negative
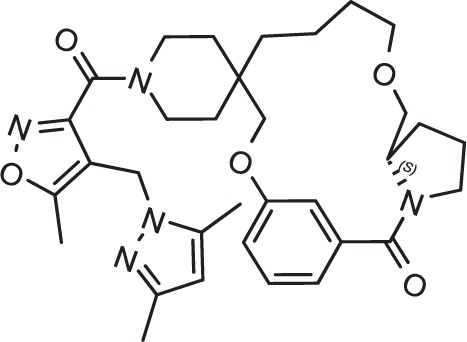

### Comparison to Other Methods

The external validation process can also be used for traditional approaches of virtual screening. As in traditional approaches, a Random Forest (RF) and a Fully Connected Neural Network (NN) models are trained on the ECFP4 of the molecules (with size 1024) after optimized hyper-parameters were found. RF is chosen since it offers a fair amount of control over over-fitting. Both models pass through the process of external validation to be given the optimum hyper-parameters. Furthermore, in order to evaluate the impact of transfer learning, a model without transferred weights is trained. The results are shown in **Table 5**.

**Table 5:** Comparison of different models on test dataset.

	Featurization	Accuracy	Recall	ROC-AUC
Random Forest	ECFP4	14.08	89.79	0.51
				
DeepMalaria without Transfer Learning	GraphConv	33.46	77.55	0.55
DeepMalaria	GraphConv	44.13	87.75	0.69

The RF and NN models predict most of the input molecules as active, resulting in an impractical model. The GCNN in DeepMalaria can outperform RF without transfer learning, showing the superiority of learnt features during training (in GCNN) to isolated feature extraction (ECFP). When transfer learning was used the model gains a noticeable boost in performance, correctly predicting more active and inactive molecules. This shows the effectiveness of DeepMalaria’s process in early hit prediction.

### Stage Specific Activity Determination of Active Scaffold

A disadvantage of phenotypic screening compared to target-based screening is that the biological target of the active compound is unknown. However, analysis of the development stages affected by these compounds and the rate of killing may provide insight into their mechanism of action. We hypothesized that macrocyclic compounds are likely target new macromolecules in the plasmodial life cycle because of their unusual standing as a bridge between small molecule inhibitors and larger biomolecules (Driggers et al., 2008). We explore this by assessing the stage-specific activity and the rate of killing of the four novel antiplasmodial scaffolds.

#### Macrocyclic Hits Inhibit Multiple *Plasmodium* Developmental Stages

**Figure 6:**
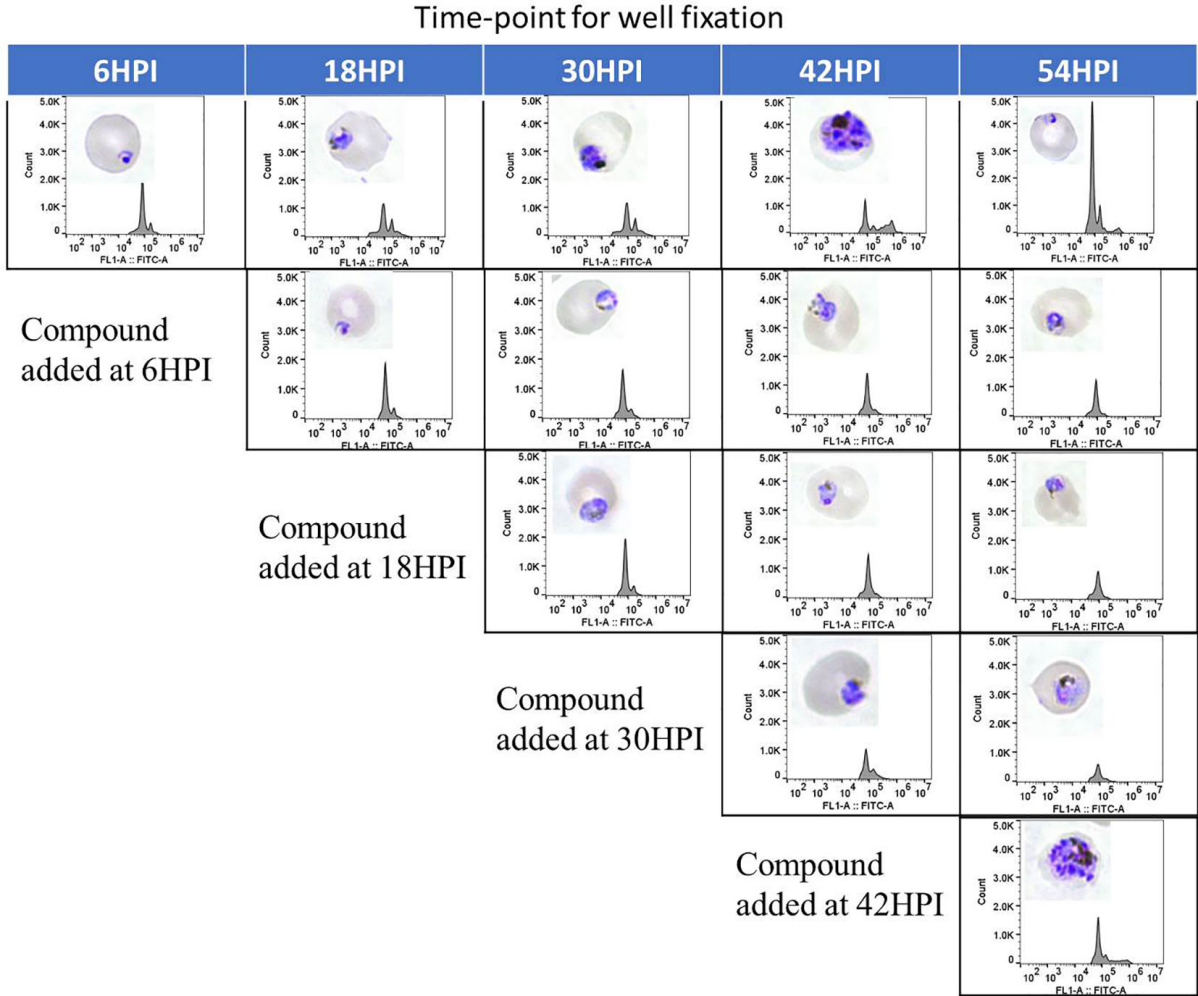
Stage Specific Activity of DC-9237. At different time-points of the malaria parasite intraerythrocytic developmental cycle, DC-9237 was added at a concentration of 3xEC_50_. Additions were at 6, 18, 30, and 42 h post-invasion (hpi). Samples were processed 12 h later and analyzed by Giemsa staining and flow cytometry of YOYO-1 stained samples.

Only few of the marketed antimalarials are able to target multiple stages of the intraerythrocytic life cycle including the early ring stage (Roberts et al., 2017). Additionally, 4 out of 12 current antimalarials, including artemisinin, inhibit growth of the early ring stage (Wilson et al., 2013). To determine the stage specific activity of the hit compounds identified in this work, synchronous culture was exposed to compounds at different time points of the life cycle and the maturation of the parasite was assessed by flowcytometric and microscopic analysis. As seen in **Figure 6**, flowcytometric and Giemsa-stained microscopic data suggest that the control culture matured as expected progressing from ring (6 h) to early trophozoite (18 h) to trophozoite (30 h) to multinucleated schizont (42 h). At 54 h parasites are at the ring stage upon reinvasion with a concomitant increase in parasitemia as evident from an increase in the flow cytometric peak. In contrast, DC-9237 inhibits all asexual blood stages of Dd2 including the early ring stage. DC-9236 inhibits primarily the mature schizonts and both DC-5921 and DC-5931 inhibit the early stages (**Supplementary Material 1**). This multistage active antiplasmodial chemotype identification from a single library is further evidence of the utility of macrocyclic compounds as candidate antimalarial scaffolds since not many compounds would target all or early asexual stages of *P. falciparum*.

#### DC-9237 Is a Fast-Acting Compound

**Figure 7:**
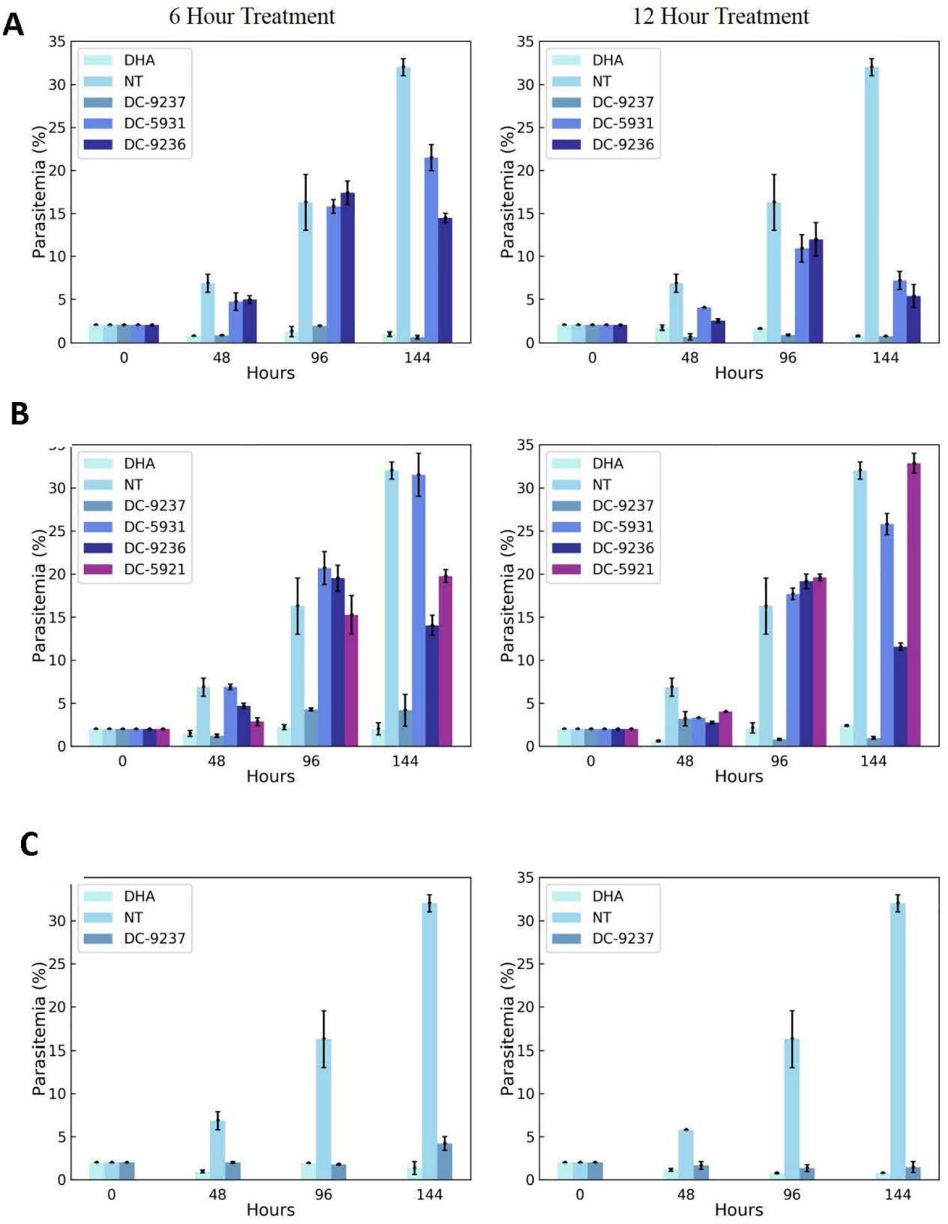
Assessment of rate of killing. Synchronous cultures were subjected to 3 x EC_50_ concentrations of macrocyclic compounds or dihydroartemisinin (DHA) for 6 or 12 h, followed by washing to remove the inhibitor and incubating in the growth medium in the absence of compounds to monitor recovery. **(A)** Compounds added at the schizont stage, **(B)** treatment at the trophozoite stage, and **(C)** ring stage culture exposed to the compound. Compounds were added at a stage where they exhibit block in cell cycle progression.

An attractive property of a successful antimalarial compound is rapid clearance of parasites, reducing the need for additional doses. Using synchronized Dd2 culture, we measured the rate of parasite killing at different stages of intraerythrocytic development. As evident from **Figure 7**, compared to the control, DC-9237 inhibited growth at all asexual stages after 6 h of exposure and parasite population did not resume growth even after 6 days. In contrast, the remaining compounds did not completely inhibit growth even at 12 h of exposure, suggesting a low rate of elimination. DC-9236 is showing higher elimination in the second life cycle. The types of compounds which are not effective at the first cell cycle, thus causing a delayed parasite death, are known to target apicoplast which is a vestigial chloroplast-like organelle (Kennedy et al., 2019b).

### Deepmalaria Identifies Drug Repurposing Candidates

**Figure 8:**
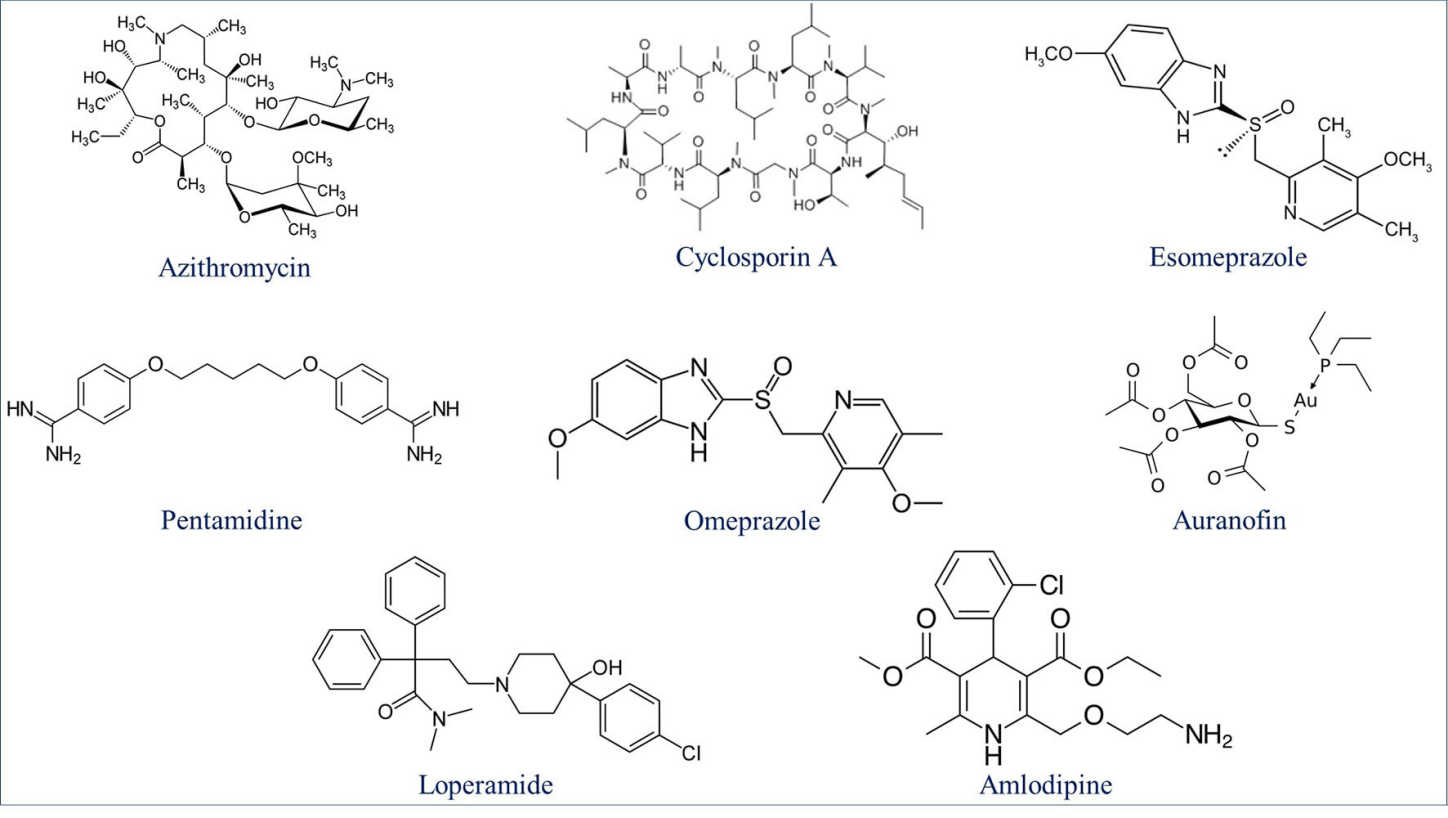
Eight drugs suggested by Pazhayam et al. (2019) with probable antimalarial activity. DeepMalaria was able to predict all scaffolds as hits for *Plasmodium falciparum*.

Drug repurposing is a very important aspect of modern drug discovery that reduces the cost significantly. Compounds that have been already approved would make suitable drugs with new medical indication since they need lower developmental costs and faster approval processes (Pushpakom et al., 2019). It has been reported that 226 FDA approved drugs are active against different stages and cell lines of *Plasmodium falciparum* (Chong et al., 2006; Derbyshire et al., 2012). After removing all inorganic molecules and also inactive ones against blood stages of Dd2 cell line, 211 drugs were screened virtually using DeepMalaria. The model showed 74% accuracy in predicting those 211 compounds as repositioning candidates (**Supplementary Material 2**). Pazhayam et al. proposed eight of those compounds as stronger candidates because of sharing common targets between the host and parasite (Pazhayam et al., 2019). These drugs include Azithromycin, Cyclosporin A, Esomeprazole, Pentamidine, Omeprazole, Auranofin, Loperamide and Amlodipine. As expected, the model predicts all of the eight candidates as potential antimalarials. This further validates the promise of DeepMalaria as a powerful tool for drug repurposing (**Figure 8**).

## Discussion

Options for malaria therapy are increasingly becoming limited because of widespread drug resistance. Even artemisinin-based combination therapies (ACTs), the front-line therapeutic choices for uncomplicated *P. falciparum* malaria, are gradually becoming ineffective in many countries of Southeast Asia (Cui, 2011; Ashley et al., 2014). Reports of failure of dihydroartemisinin-piperaquine drug combination therapy in Cambodia leaves us with very few therapeutic choices (Saunders et al., 2014). This bleak situation emphasizes the urgent need to develop new antimalarials that act on novel targets. Although recent increase in novel antimalarial discovery efforts has led to quite a few lead compounds in preclinical development (Ashley and Phyo, 2018), the need for new antimalarial drugs will continue to exist because of expected loss of new drugs due to future emergence of resistance.

In this work, a deep learning model was trained on publicly available data to predict *Plasmodium falciparum* inhibition of compounds. A validation dataset was created from previous experiments and was augmented to assist in hyper-parameter optimization. Transfer learning from a large corpus of unrelated data was employed to facilitate the training of the deep learning model. The model was tested on an independent macrocyclic test dataset in order to find new drug candidates. DeepMalaria was able to find 72.32% of active molecules from the validation dataset and 87.75% of that of the test dataset, while maintaining an acceptable accuracy in an imbalanced setting. The results show that deep learning automatic feature extraction can learn patterns within the molecules that are generalizable to new and unseen datasets, outperforming the traditional approach of classifying fingerprints. DeepMalaria has shown increasing accuracy when predicting more potent compounds, a very important characteristic which did not let any nanomolar active/non-cytotoxic compound to be missed. Furthermore, the hit compounds were narrowed down to one fast-acting compounds working at all stages of *P. falciparum* growth. Also, DC-9236 showed inhibition in the second developmental cycle of Pf causing delayed death most likely because of its action on the apicoplast. Compounds with delayed death characteristics would be a very good candidate for combination therapy (Kennedy et al., 2019a).

We demonstrated the potential of deep learning and the Transilico architecture to accelerate the process of active compound identification in early drug discovery (www.transilico.com). Since last decade AI and especially deep learning is generating new hope in small molecule early drug discovery (Leelananda and Lindert, 2016). There is an increased interest to use machine learning and related technologies to rapidly discover novel pharmacophores thus avoiding the expense of HTS (Fleming, 2018). Given the pressing need for novel antimalarials, accelerated hit identification through the use of AI as has been presented in this work would be of great interest. Artificial intelligence has revolutionized many fields of medicine including drug discovery (Wang and Shen, 2017; Doan and Carpenter, 2019; Topol, 2019). Expensive HTS, low hit rate of synthetic libraries, incompatibility of natural products with HTS, non-diverse libraries etc., are some of the reasons for limited success of many of today’s drug discovery efforts (Koehn and Carter, 2005; Li and Vederas, 2009; Schneider, 2017). However, many AI-based approaches of early drug discovery such as structural based VS and de novo design of molecules are still relatively unexplored in malaria therapeutics development. After the success of abstract and superior performance of deep learning feature extraction, Generative Adversarial Networks and Variational Auto-Encoders would act as good candidates for leveraging this abstract representation for bioactive molecule identification.

To establish AI for accelerated malaria drug lead discovery, we used a commercial macrocyclic compound library for validation. Peptidic macrocycles compounds have characteristics of both small molecules and polypeptides, and have not been investigated for antimalarial therapeutics discovery. High hit rate of macrocyclic compound screening suggests their utility as antimalarials. Other classes of macrocyclic compounds such as cyclic peptides would also be a good candidate for further study (Whitty et al., 2017). It is of note that, although the model was trained on a synthetic library, DeepMalaria was highly accurate in discovering natural products hits. This article is the first report regarding the use of transfer learning for malaria drug discovery and will be a model for future projects of AI-based drug discovery. Additionally, our model would aid in drug repurposing as it showed strength in predicting potential antimalarial activities of already approved drugs.

## Data Availability Statement

All datasets generated for this study are included in the article/**Supplementary Material**. The code for this work can be accessed through www.transilico.com.

## Author Contributions

AA conducted all biological experiments, acquired data for Deep Malaria, optimized the *in-silico* results, and wrote the relevant parts of the manuscript. MS trained and optimized the *in silico* model and wrote the relevant parts of the manuscript. JC screened most of evaluation data. DC provided guidance for biological experiments for malaria and DeepMalaria data acquisition. JY provided guidance in the opportunities of deep learning in a multidiscipline collaboration. All authors have read the submitted manuscript.

## Funding

Funding for this research is from intramural funding (University of Central Florida, College of Medicine).

## Conflict of Interest

The authors declare that the research was conducted in the absence of any commercial or financial relationships that could be construed as a potential conflict of interest.
