# Supplementary material for: DeepMalaria: Artificial Intelligence Driven Discovery of Potent Antiplasmodials
Source: Front Pharmacol. 2020 Jan 15;10:1526. doi: 10.3389/fphar.2019.01526 (PMC6974622; doi:10.3389/fphar.2019.01526)
Supplement: Supplementary file 1 [file DataSheet_1.docx]

Supplementary Material

# Supplementary In silico Results

## DeepMalaria Training Results

The results of training DeepMalaria model is shown in Supplementary Figure 1.


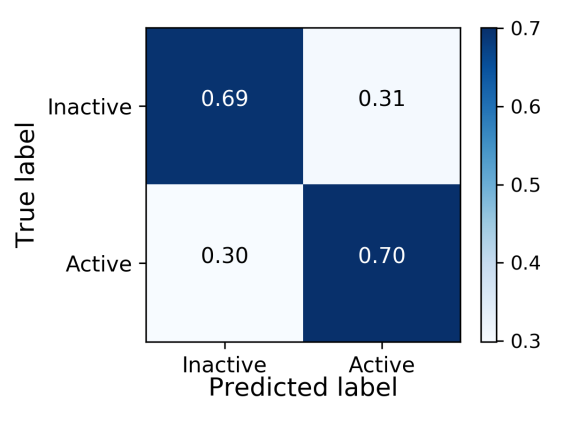


**Supplementary Figure 1.** The confusion matrix of DeepMalaria model on training dataset.

## ECFP Hyper-Parameters and Results

Grid search is applied on the RF model in order to find the optimum hyper-parameters. The chosen parameters are shown in Supplementary Table 1.

Supplementary Table 1. Optimum hyper-parameters of the RF model.

| **Hyper-Parameter** | **Optimum Value** | **Hyper-Parameter** | **Optimum Value** |
| --- | --- | --- | --- |
| # of Estimators | 200 | Max Depth | 7 |
| Min Sample Leaf | 10 | Max Features | 0.3 |

The hyper-parameters are sensible since a high number of estimators using a small amount of features can help avoid over-fitting. The confusion matrices of training, validation, and test datasets are shown in Supplementary Figure 2.


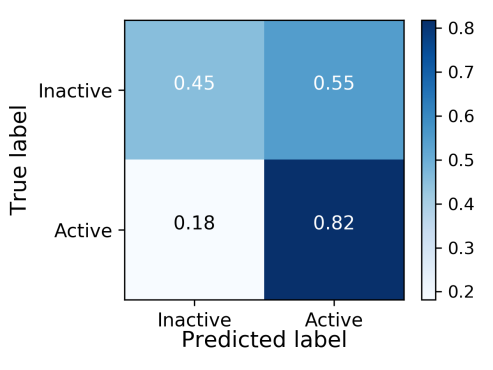

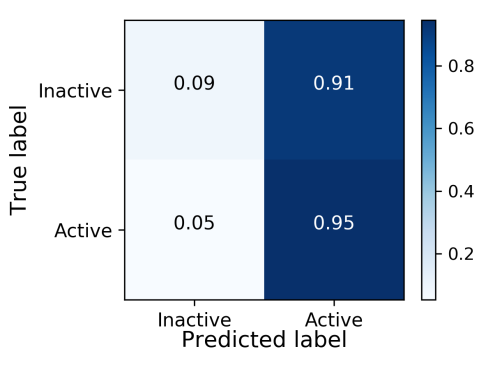

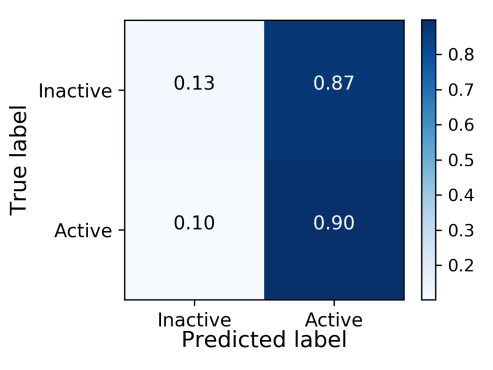


**Supplementary Figure 2.** The confusion matrices of RF model on training dataset (left), validation dataset (center), and test dataset (right).

This process is also repeated using a Fully Connected Neural Network (NN). The optimum parameters are shown in Supplementary Table 2. Adam optimizer is used with 0.9 momentum.

Supplementary Table 2. Optimum hyper-parameters of the NN model.

| **Hyper-Parameter** | **Optimum Value** | **Hyper-Parameter** | **Optimum Value** |
| --- | --- | --- | --- |
| Number of hidden layers | 1 | Initial learning rate | 0.1 |
| Hidden layer size | 8 | Maximum iterations | 1000 |

Different number of hidden layers (1, 2, and 3) were chosen for the hyper-parameter optimization, however, the external validation found that 1 layer with 8 neurons is the optimum architecture.

## GCNN Architecture Offered by DeepChem

The DeepChem Library offers creation of Graph Convolutional Networks using a featurization layer, graph convolution layer(s) followed by batch normalization, dropout, and graph pooling. The intermediate outputs are then passed to a fully connected layer, again followed by batch normalization and dropout. Consequently, the neural fingerprint is then created from the output of a graph gather layer. This abstract fingerprint in then classified using one fully connected layer. In this work, as stated by table 2, the number of convolution layers, the size of each layer, the number of neurons in the fully connected networks, the dropout rate, the learning rate, and the batch size are changed during external validation.

## DeepMalaria without Transfer Learning Results

DeepMalaria uses transfer learning to pre-train the GCNN. In this work, a model is also trained without transfer learning using the same parameters and process of DeepMalaria. The results of training this model on the training dataset is show in Supplementary Figure 3.


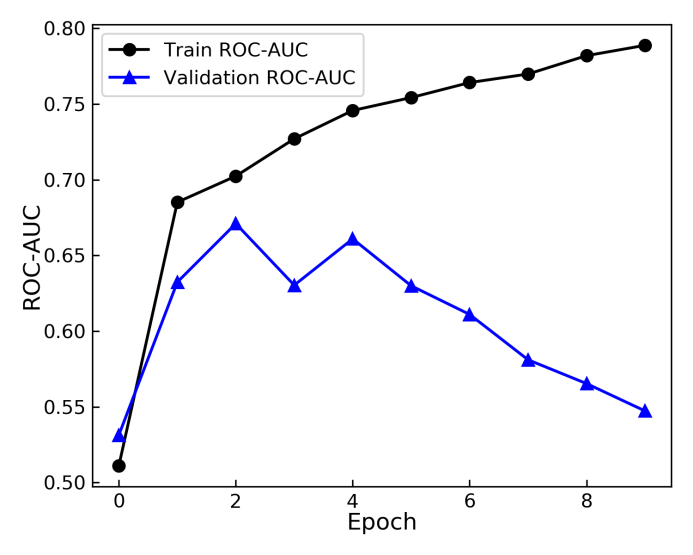


**Supplementary Figure 3.** AUC scores of the model during training without transfer learning. The model is evaluated on the training and augmented validation dataset at the end of each epoch. The model starts to over-fit after 2 epochs.

Compared to training with transfer learning (Figure 3) the starting AUC at epoch 0 and the maximum AUC on the training dataset on epoch 9 have decreased. Therefore, transfer learning allows the model to learn more useful patterns from the training dataset. The model still over-fits at the second epoch. The confusion matrices of the model without transfer learning are shown in Supplementary Figure 4.


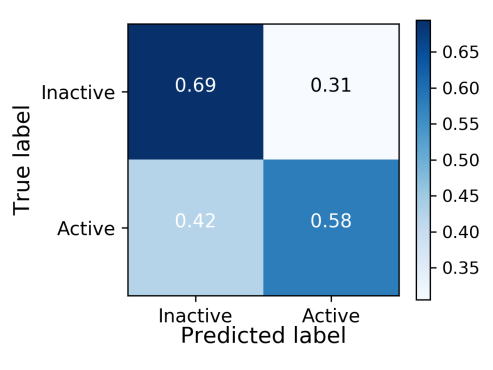

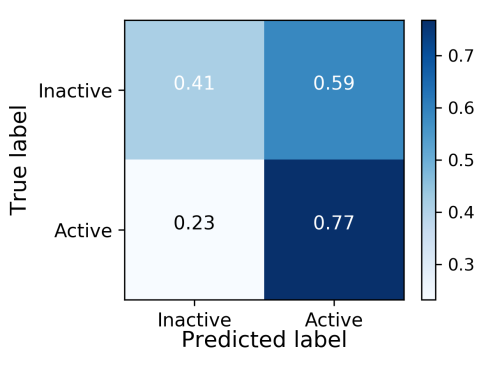

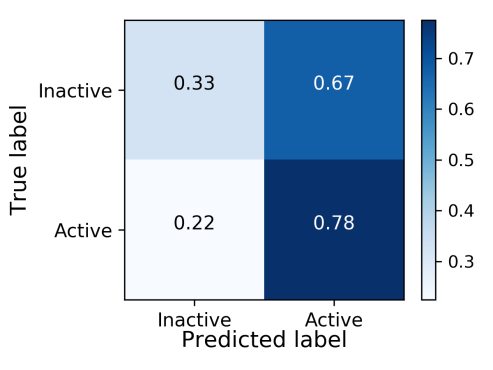


**Supplementary Figure 4.** The confusion matrices of DeepMalaria model without transfer learning on training dataset (left), validation dataset (center), and test dataset (right).

Via comparing these results to Figure 4 and Supplementary Figure 4, it can be seen that transfer learning benefitted learning from the training dataset and performing accurately on the validation and test sets.

# Supplementary In Vitro Results


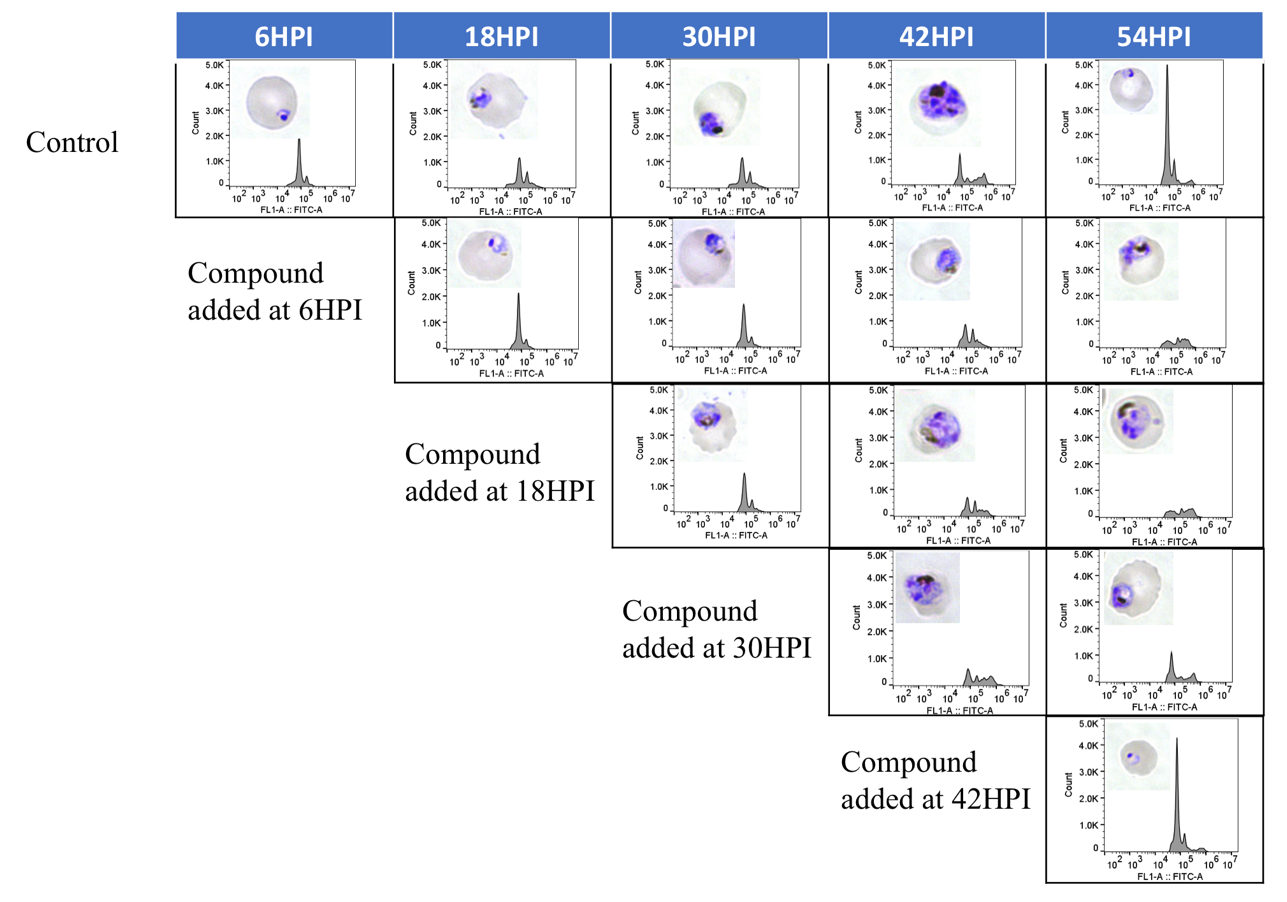


Compound DC-9236


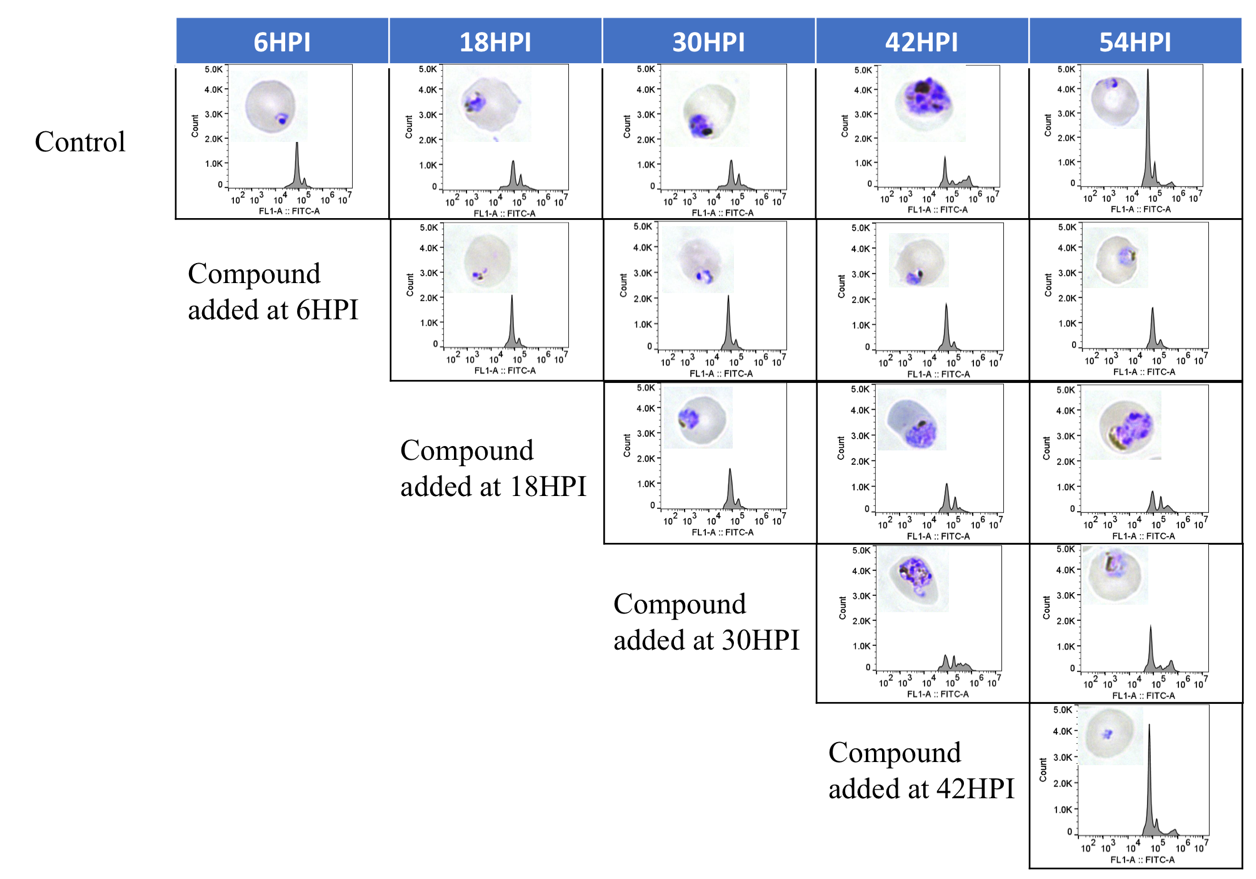


Compound DC-5931


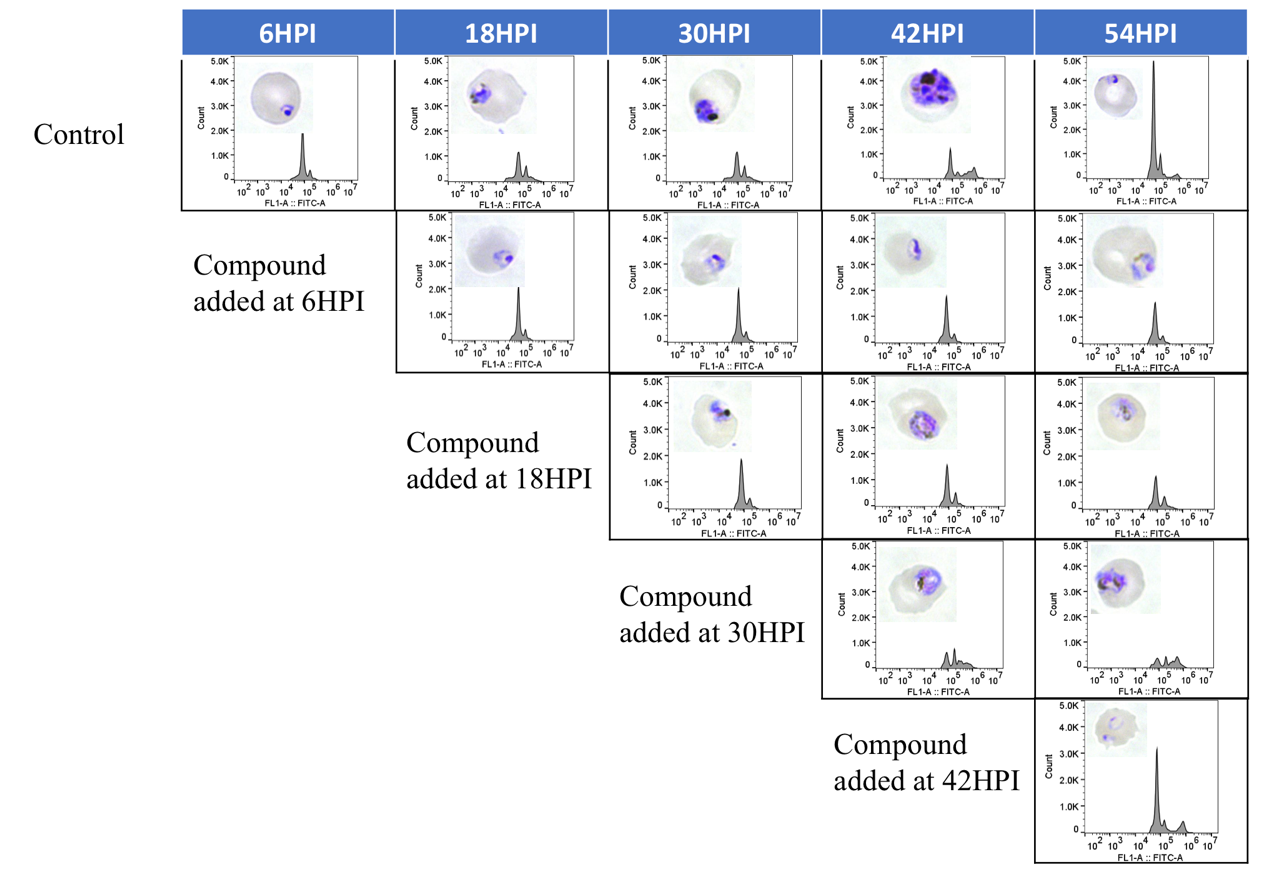


Compound DC-5921

**Supplementary Figure 5.** Stage Specific Activity for three of hits. DC-5931 is mostly active in early stages. DC-5921 is active in early stages, late Troph and early schizont. DC-9236 is active in late Troph and early Schizont.

**3 Smiles for seven hits:**

**DC-9239:** CN1CCOC2=C(CCCCCC3(CCN(CC3)C(=O)[C@H](N)C3=CC=CC=C3)C1=O)C=CC=C2

**DC-9235:** CN1CCOC2=C(CCCCCC3(CCN(CC3)C(=O)[C@@H](N)C3=CC=CC=C3)C1=O)C=CC=C2

**DC-9236:** CC(C)C[C@@H](N)C(=O)N1CCC2(CC1)CCCCCC1=C(OCCN(C)C2=O)C=CC=C1

**DC-9237:** CCC1N(CC2=CC=CC=C2OCCN(C)C)CCCCN2C=C(CN(CC3=CC=CC=C3)C3=C(CC4=CC=CC=C4)N=C1O3)N=N2

**DC-5931:** CCC1=NC2=C(C=CC=C2)C(C(=O)N2CCC3(CC2)CCCCOCCNC(=O)C2=C(OC3)C=CC=C2)=C1C

**DC-5921:** CC1=NN(CC2=C(C)ON=C2C(=O)N2CCC3(CC2)CCCCOC[C@@H]2CCCN2C(=O)C2=CC(OC3)=CC=C2)C(C)=C1

**DC-2506:** C[C@@H]1NC(=O)C2(CCCC2)NC(=O)CC[C@H](NC(=O)C2=CC=CC=C2OC[C@H](CC2=CC=CC=C2)N(C)C1=O)C(=O)NCC1=CC=CC(CN2CCCCCC2)=C1
